# Chemical composition analysis and *in vitro* biological activities of ten essential oils in human skin cells

**DOI:** 10.1016/j.biopen.2017.04.001

**Published:** 2017-04-26

**Authors:** Xuesheng Han, Cody Beaumont, Nicole Stevens

**Affiliations:** dōTERRA International, LLC, 389 S. 1300 W., Pleasant Grove, UT 84062, USA

**Keywords:** Anti-proliferation, Bergamot, Cilantro, Anti-inflammatory, Spikenard, Wound healing

## Abstract

Research on the biological effects of essential oils on human skin cells is scarce. In the current study, we primarily explored the biological activities of 10 essential oils (nine single and one blend) in a pre-inflamed human dermal fibroblast system that simulated chronic inflammation. We measured levels of proteins critical for inflammation, immune responses, and tissue-remodeling processes. The nine single oils were distilled from *Citrus bergamia* (bergamot), *Coriandrum sativum* (cilantro), *Pelargonium graveolens* (geranium), *Helichrysum italicum* (helichrysum), *Pogostemon cablin* (patchouli), *Citrus aurantium* (petitgrain), *Santalum album* (sandalwood), *Nardostachys jatamansi* (spikenard), and *Cananga odorata* (ylang ylang). The essential oil blend (commercial name Immortelle) is composed of oils from frankincense, Hawaiian sandalwood, lavender, myrrh, helichrysum, and rose. All the studied oils were significantly anti-proliferative against these cells. Furthermore, bergamot, cilantro, and spikenard essential oils primarily inhibited protein molecules related to inflammation, immune responses, and tissue-remodeling processes, suggesting they have anti-inflammatory and wound healing properties. Helichrysum and ylang ylang essential oils, as well as Immortelle primarily inhibited tissue remodeling-related proteins, suggesting a wound healing property. The data are consistent with the results of existing studies examining these oils in other models and suggest that the studied oils may be promising therapeutic candidates. Further research into their biological mechanisms of action is recommended. The differential effects of these essential oils suggest that they exert activities by different mechanisms or pathways, warranting further investigation. The chemical composition of these oils was analyzed using gas chromatography–mass spectrometry.

## Highlights

• Biological effects of 10 essential oils were examined in human dermal fibroblasts.
• All investigated oils showed significant anti-proliferative effects in fibroblasts.
• Bergamot, cilantro, and spikenard oils primarily showed anti-inflammatory effects.
• Essential oils showed differential effects, likely mediated by different mechanisms.

## 1 Introduction

Essential oils (EOs) are naturally occurring aromatic molecules in plants. EOs have a long history of traditional use and have specifically gained popularity for skincare purposes. However, scientific studies of the biological effects of EOs on human skin cells are very scarce. Recent studies of EOs in human dermal fibroblasts showed that they robustly affect proteins, genes, and pathways related to inflammation and tissue-remodeling processes [1], [2], [3].

In this study, we explored the biological activities of 10 EOs in a previously described human dermal fibroblast system [4], [5]. The 10 EOs were distilled from *Citrus bergamia* (bergamot), *Coriandrum sativum* (cilantro), *Pelargonium graveolens* (geranium), *Helichrysum italicum* (helichrysum), *Pogostemon cablin* (patchouli), *Citrus aurantium* (petitgrain), *Santalum album* (sandalwood), *Nardostachys jatamansi* (spikenard), and *Cananga odorata* (ylang ylang), as well as Immortelle, an EO blend composed of frankincense, Hawaiian sandalwood, lavender, myrrh, helichrysum, and rose oils.

## 2 Materials and methods

All experiments were conducted using a Biologically Multiplexed Activity Profiling (BioMAP) system HDF3CGF, which was designed to model the pathology of chronic inflammation in a robust and reproducible manner. The system is comprised of a cell type, molecular stimuli to recreate the disease environment, and a set of biomarker (protein) readouts to examine treatment effects on the disease environment [5]. The methodologies used in this study were essentially the same as those previously described [5], [6].

### 2.1 Essential oils

Each EO (dōTERRA Intl., Pleasant Grove, UT, USA) was diluted in dimethyl sulfoxide (DMSO) to 8 × the specified concentration (final DMSO concentration in culture media was no more than 0.1% [v/v]). Then, 25 μl of each 8 × solution was added to the cell culture to obtain a final volume of 200 μl; DMSO (0.1%) served as the vehicle control.

The chemical composition of the EOs was analyzed using gas chromatography–mass spectrometry (GC–MS). GC–MS analysis of the EOs was conducted using a gaseous chromatograph (Agilent Technologies 6890N) with mass detector (5973 Network) equipped with an automatic sample injector (7683 Series Injector). Sample preparation consisted of dissolving 0.02 ml of analyzed EO in 1.0 ml Hexane. Each sample injection was repeated three times. A DB-5MS capillary column 30 cm in length, with a diameter of 0.25 mm was used. The stationary phase film thickness was 0.25 μm, and the flow rate of the carrier gas nitrogen was 1.2 ml/min. The temperature of the injector was 250 °C, ion source temperature was 230 °C, and the temperature of the quadrupole was 150 °C. Samples (3 μl) were injected in a split mode (40:1). The analyses were carried out in a scan mode in an *m*/*z* range of 40–500. Time of analyte allocation was 52 min. The column temperature program was 40 °C for 3 min, followed by 80 °C for 2 min, 120 °C for 5 min, 200 °C for 2 min, and 250 °C for another 2 min. ChemStation software was used to collect and process the data. Identification of compounds in samples was conducted by comparing MS spectra with standard spectra from the NIST 2014 library; retention indices were determined. The compounds showing conformity of mass spectra with the standard library spectra of more than 95% were considered. To confirm the identification, retention indices of the analyzed compounds were compared with the literature data. Relative percentage content of the analyzed compounds was based on the peak area of the total ionic current of all compounds present in each sample.

### 2.2 Cell culture

Primary human neonatal fibroblasts were prepared as previously described [7] and were plated under low serum conditions (0.125% fetal bovine serum) for 24 h. Then the cell culture was stimulated with a mixture of interleukin (IL)-1β, tumor necrosis factor (TNF)-α, interferon (IFN)-γ, basic fibroblast growth factor (bFGF), epidermal growth factor (EGF), and platelet-derived growth factor (PDGF), for another 24 h. The cell culture and stimulation conditions for the HDF3CGF assays have been described in detail elsewhere, and the culture was performed in a 96-well plate [7], [8].

### 2.3 Protein-based readouts

An enzyme-linked immunosorbent assay (ELISA) was used to measure biomarker levels of cell-associated and cell membrane targets. Soluble factors in the supernatants were quantified using homogeneous time-resolved fluorescence detection, bead-based multiplex immunoassay, or capture ELISA. Adverse effects of the test agents on cell proliferation and viability (cytotoxicity) were measured using the sulforhodamine B (SRB) assay. For proliferation assays, cells were cultured and examined after 72 h, which is optimal for the HDF3CGF system; the detailed procedure has been previously described [7]. Measurements were performed in triplicate wells; a glossary of the biomarkers used in this study is provided in Supplementary Table S1.

### 2.4 Statistical analysis

Quantitative biomarker data are presented as the mean log_10_ relative expression level (compared to the respective mean vehicle control value) ± standard deviation (SD) of triplicate measurements. Differences in biomarker levels between EO- and vehicle-treated cultures were tested for significance using an unpaired student's *t*-test. A *p* < 0.01 outside the significance level with an effect of at least 10% (more than 0.1 log_10_ ratio units) was considered significant.

## 3 Results

The primary chemical components (i.e., >2%) of the tested EOs are listed in Table 1. Table S1 in Supplementary material shows a glossary of the biomarkers analyzed in this study. These included the inflammation-related protein molecules monocyte chemoattractant protein 1 (MCP-1), vascular cell adhesion molecule 1 (VCAM-1), intracellular cell adhesion molecule 1 (ICAM-1), interferon gamma-induced protein 10 (IP-10); interferon-inducible T-cell alpha chemoattractant (I-TAC), IL-8, and monokine induced by gamma interferon (MIG); tissue remodeling-related protein molecules collagen I and III, epidermal growth factor receptor (EGFR), matrix metalloproteinase 1 (MMP-1), plasminogen activator inhibitor 1 (PAI-1), and tissue inhibitor of metalloproteinase (TIMP) 1 and 2; and the immunomodulation-related protein molecule, macrophage colony-stimulating factor (M-CSF). Cell proliferation and viability were measured using an SRB assay.

**Table 1:** Primary chemical components (i.e., >2.0%) of the studied ten essential oils.

Essential oil	Chemical components (%)
Bergamot	Limonene (35.8), Linalyl Acetate (33.0), gamma-Terpinene (9.0), Linalool (7.5), beta-Pinene (7.0)
Cilantro	trans-2-Decenal (30.0), trans-2-Decen-1-ol (15.8), Linalool (15.5), trans-2-Dodecenal (7.0), Decanal (4.7), Decanol (4.6), Tetradecanol (3.0), gamma-Terpinene (2.2)
Geranium	Citronellol (38.3), Citronellyl formate (11.6), 6,9-Guanidiene (7.4), Geraniol (6.4), Menthone (3.3), Isomenthone (3.3), Linalool (3.3), cis-Rose Oxide (2.0)
Helichrysum	Neryl acetate (35.5), gamma-Curcumene (13.9), alpha-Pinene (8.9), alpha-Curcumene (4.3), Italicene (4.0), Limonene (3.6), Neryl Propionate (3.1), Eucalyptol (2.3), Copaene (2.2)
Patchouli	Patchoulol (39.6), alpha-Bulnesene (19.1), Aromadendrene (17.8), alpha-Guaiene (13.4), alpha-Patchoulene (4.8), Aciphyllene (2.7), beta-Patchoulene (2.7), beta-Caryophyllene (2.3)
Petitgrain	Linalyl acetate (50.3), Linalool (23.9), alpha-Terpineol (6.7), Geranyl Acetate (4.1), trans-beta-Ocimene (2.3), Neryl Acetate (2.5), Myrcene (2.0)
Sandalwood	cis-alpha-Santalol (48.4), cis-beta-Santalol (22.0), cis-alpha-trans-Bergamotol (6.8), cis-epi-beta-Santalol (3.9), cis-Lanceol (3.2), cis-Nuciferal (2.0), beta-Santalene (2.0)
Spikenard	beta-Gurjunene (11.3), Jatamansone (9.5), Spirojatamol (8.7), 6,9-Guanidiene (7.5), Valencene (5.6), Valerena-4,7(11)-diene (5.6), Seychellene (3.8), 7-epi-alpha-Selinene (2.9), gamma-Vetivenene (2.6), beta-Pinene (2.5), alpha-Aristolenol (2.4), (−)-alpha-Gurjunene (2.2), beta-Patchoulene (2.0)
Ylang Ylang	Germacrene D (23.2), beta-Caryophyllene (14.7), Geranyl Acetate (6.4), Benzyl Benzoate (6.1), Linalool (5.1), delta-Cadinene (4.3), alpha-Humulene (3.9), para-methyl-Anisole (2.9)
Immortelle blend	alpha-Pinene (23.1), cis-alpha-Santalol (10.2), Limonene (6.8), Linalyl Acetate (6.3), Linalool (4.7), cis-beta-Santalol (4.4), alpha-Thujene (4.1), beta-Caryophyllene (3.8), Curzerene (3.6), Octyl Acetate (3.3), beta-Pinene (3.1), Furanoeudesma-1,3-diene (2.5), Sabinene (2.0)

**Fig. 1:**
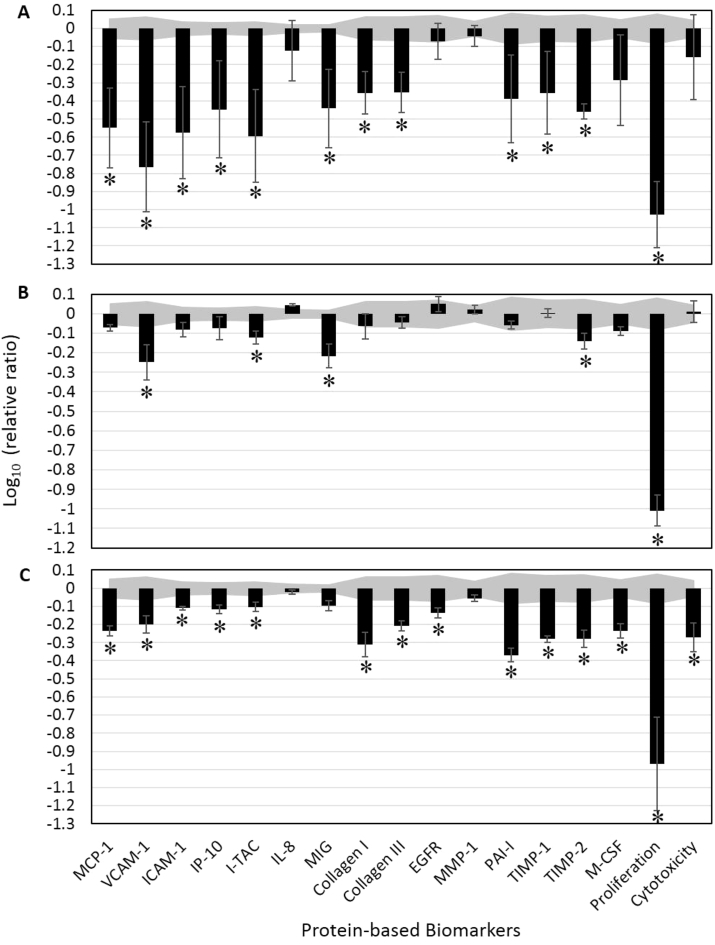
Bioactivity profiles of essential oils (EOs), including (A) bergamot (0.01% v/v), (B) cilantro (0.0011%), and (C) spikenard (0.0033%) in a human dermal fibroblast culture (HDF3CGF). The *Y*-axis denotes the relative expression levels of biomarkers compared to vehicle control values in log_10_ form. Vehicle control values are shaded in gray, denoting the 95% significance level. Error bars represent the standard deviations from triplicate measurements. *Biomarkers designated with “key activity,” i.e., significantly different (*p* < 0.01) from vehicle controls, outside the significance level, with an effect of at least 20% (more than 0.1 log ratio units). MCP-1, monocyte chemoattractant protein; VCAM-1, vascular cell adhesion molecule 1; ICAM-1, intracellular cell adhesion molecule 1; IP-10, interferon gamma-induced protein 10; I-TAC, interferon-inducible T-cell alpha chemoattractant; IL-8, interleukin-8; MIG, monokine induced by gamma interferon; EGFR, epidermal growth factor; MMP-1, matrix metalloproteinase 1; PAI-1, plasminogen activator inhibitor 1; TIMP, tissue inhibitor of metalloproteinase; M-CSF, macrophage colony-stimulating factor.

The effects of four concentrations (0.01, 0.0033, 0.0011, and 0.00037%, v/v) of each EO on cell viability were initially studied. The highest concentration of each oil that was not overtly cytotoxic (log_10_ relative ratio ≤ −0.3) to these cells was further analyzed, as discussed below. Key biomarker modulation was inferred when the biomarker values in the treated cells were significantly different (*p* < 0.01) from those of the vehicle controls, with an effect of at least 20% (more than 0.1 log ratio units) (Fig. 1).

### 3.1 Effects of bergamot, cilantro, and spikenard EOs in human dermal fibroblasts

Overall, the bergamot EO (0.01%) inhibited all 17 analyzed biomarkers (Fig. 1A). Specifically, it significantly inhibited the inflammatory molecules, MCP-1, VCAM-1, ICAM-1, IP-10, I-TAC, and MIG, as well as the tissue remodeling-related molecules, collagen I and III, PAI-1, and TIMP-1 and TIMP-2. Bergamot EO also showed a strong and significant anti-proliferative activity in these cells. Bergamot EO did not significantly affect IL-8, EGFR, MMP-1, or cytotoxicity. Of note, bergamot EO strongly inhibited the level of M-CSF, nearly reaching significance (0.05 > *p* > 0.01).

Cilantro EO (0.0011%) significantly inhibited cell proliferation (Fig. 1B) as well as the production of three inflammatory biomarkers VCAM-1, I-TAC, and MIG, and one tissue-remodeling biomarker, TIMP-2. Cilantro EO did not significantly affect the level of other analyzed biomarkers. Spikenard EO (0.0033%) inhibited the majority of studied biomarkers (Fig. 1C). Specifically, it significantly inhibited the inflammatory biomarkers MCP-1, VCAM-1, ICAM-1, IP-10, and I-TAC, but not IL-8 or MIG. Spikenard EO also significantly inhibited the tissue-remodeling molecules collagen I and III, EGFR, PAI-1, and TIMP-1 and TIMP-2, as well as the immunomodulatory molecule M-CSF. Spikenard EO showed significant anti-proliferative and cytotoxic activity, although the toxicity was not considered overt (log_10_ relative ratio ≤ −0.3).

### 3.2 Effects of helichrysum, ylang ylang, Immortelle, and other EOs in human dermal fibroblasts

**Fig. 2:**
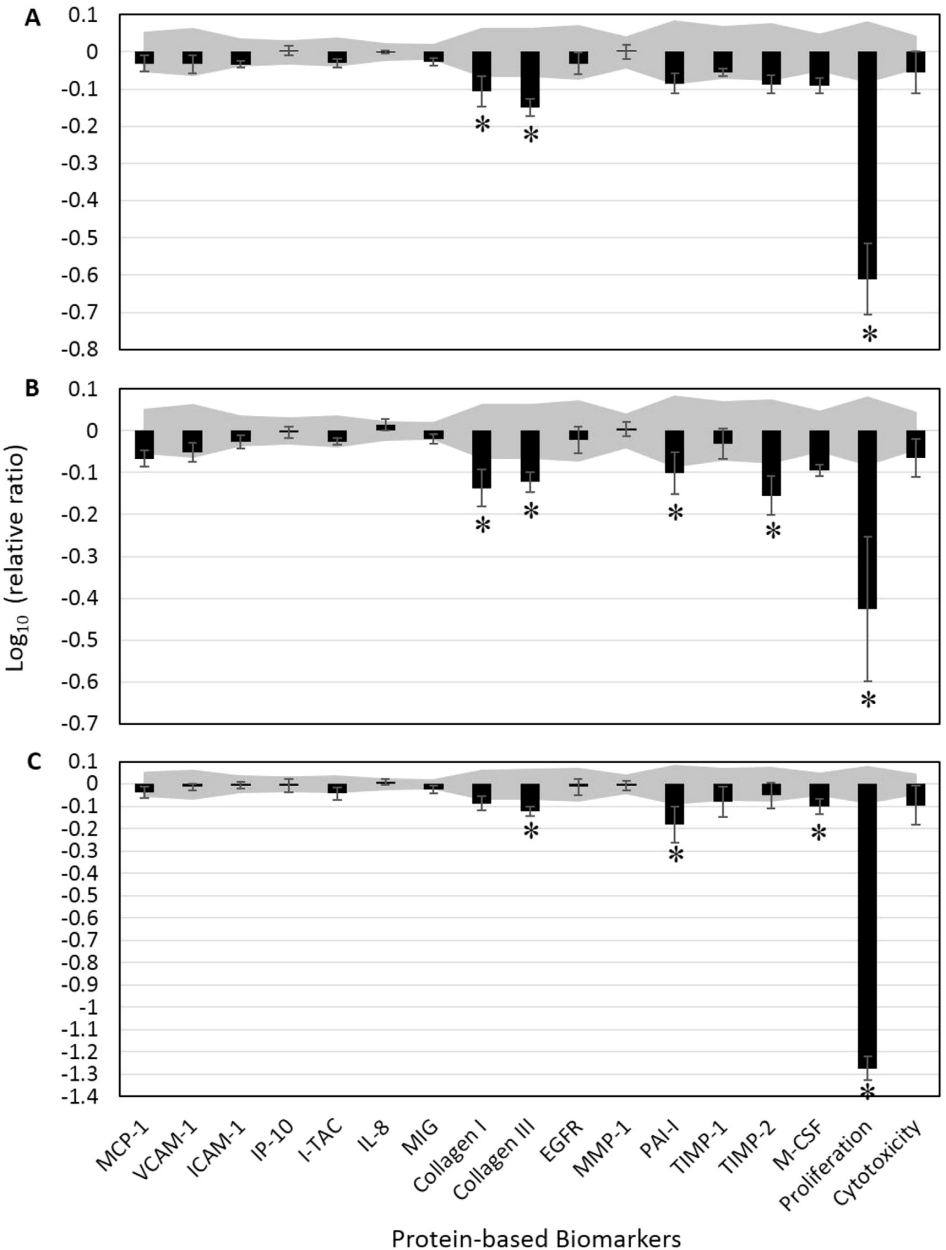
Bioactivity profiles of essential oils (EOs), including (A) helichrysum (0.01% v/v), (B) ylang ylang (0.0033%), and (C) Immortelle (0.0033%) in a human dermal fibroblast culture (HDF3CGF). The *Y*-axis denotes the relative expression levels of biomarkers compared to vehicle control values in log_10_ form. Vehicle control values are shaded in gray, denoting the 95% significance level. Error bars represent the standard deviations from triplicate measurements. *Biomarkers designated with “key activity,” i.e., significantly different (*p* < 0.01) from vehicle controls, outside the significance level, with an effect of at least 20% (more than 0.1 log ratio units). MCP-1, monocyte chemoattractant protein; VCAM-1, vascular cell adhesion molecule 1; ICAM-1, intracellular cell adhesion molecule 1; IP-10, interferon gamma-induced protein 10; I-TAC, interferon-inducible T-cell alpha chemoattractant; IL-8, interleukin 8; MIG, monokine induced by gamma interferon; EGFR, epidermal growth factor; MMP-1, matrix metalloproteinase 1; PAI-1, plasminogen activator inhibitor 1; TIMP, tissue inhibitor of metalloproteinase; M-CSF, macrophage colony-stimulating factor.

Three of the studied EOs significantly inhibited some tissue-remodeling molecules, but not the inflammatory molecules (Fig. 2). Specifically, helichrysum EO (0.01%) significantly inhibited the production of collagen I and III; ylang ylang EO (0.0033%) significantly inhibited collagen I, III, PAI-1, and TIMP-2; and Immortelle (0.0033%) significantly inhibited collagen III and PAI-1. Immortelle also slightly inhibited the production of M-CSF, an immunomodulatory molecule. All three of these EOs showed significant anti-proliferative effects.

Four other studied EOs (geranium, 0.01%; patchouli, 0.0033%; petitgrain, 0.01%; and sandalwood, 0.0011%) were found to be significantly anti-proliferative (data not shown). Patchouli EO also significantly inhibited the production of PAI-1; however, none of these four oils significantly affected the other analyzed biomarkers.

## 4 Discussion

The protein-based biomarker analysis showed differential effects of these 10 EOs in a pre-inflamed human dermal fibroblast system. Bergamot, cilantro, and spikenard EOs significantly inhibited some inflammatory, tissue remodeling, and immunomodulatory biomarkers, suggesting anti-inflammatory, immunomodulatory, and wound healing properties. Helichrysum, ylang ylang, and Immortelle EOs significantly inhibited several tissue-remodeling molecules, suggesting promising wound healing potential. All the studied EOs showed strong and significant anti-proliferative activity. These findings indicate that these EOs are biologically active in human skin cells, and suggest they may exert their activities via different mechanisms of action.

Bergamot oil and its major active components, namely limonene, linalyl acetate, and linalool, have demonstrated anti-inflammatory, immunomodulatory, and wound healing activities under different conditions [9], [10], [11]. Cilantro oil and its major active component, linalool, have also been reported to possess anti-inflammatory and wound healing properties [12], [13]. Of note, a literature search revealed no published studies of spikenard or its major active components in human cells or their anti-inflammatory and wound healing activities. However, mouse studies [14], [15] have reported that inhalation of spikenard extract has a significant sedative effect, which might be partially attributed to its anti-inflammatory activity. To the best of our knowledge, the current study provides the first evidence of the anti-inflammatory, immunomodulatory, and wound-healing properties of spikenard oil. Further comprehensive investigations of the biological and pharmacological mechanisms of action of spikenard EO are recommended.

Helichrysum EO has been extensively studied for its antibacterial and antifungal activities [16], [17], which might be involved in its tissue remodeling and anti-proliferative effects and thus contribute to its role in the wound healing process. Ylang ylang EO has been shown to possess antibacterial, antifungal, antioxidant, and anti-inflammatory properties [18]. One major EO found in Immortelle, frankincense, has been reported to inhibit collagen III, IP-10, and ICAM-1 in the same cell culture system used in the current study [19]. The observed inhibitory effect of Immortelle EO on tissue-remodeling and immunomodulatory mediators in the highly inflamed skin cells might be largely attributable to its content of alpha-pinene, linalyl acetate, linalool, and other components [20].

Studies have demonstrated the anti-inflammatory and immunomodulatory activities of geranium EO [21], [22], antifungal activity of patchouli EO [23], antimicrobial and antioxidant activities of petitgrain EO [24], [25], and the anticancer and anti-proliferative activities of sandalwood EO [26], [27]. The observed robust, anti-proliferative activity of these EOs in human dermal fibroblasts in this study seems consistent with those of existing studies.

Together, these results suggest that the studied EOs may exert beneficial effects on the human skin. Further research such as investigations of the mechanisms of action as well as the clinical efficacy and safety of these EOs may enhance our understanding of their potential therapeutic benefits. Clinical trials of EOs have been extensively focused on the efficacy of aromatherapy for mental health. For instance, a clinical trial [28] demonstrated that aroma inhalation of geranium EO effectively reduces anxiety during childbirth. More recently, a 15-min exposure to bergamot aroma was shown to improve the positive feelings of patients in a mental health treatment center [29].

Results of this current *in vitro* study in human skin cells cannot be directly extrapolated to more complex human systems. Nevertheless, this study provides important evidence for the biological activities of these studied EOs in pre-inflamed human skin cells. The differential effects of these studied EOs suggest that they may exert activities via different mechanisms, warranting further investigation.

## 5 Conclusion

This study investigated the biological activities of 10 EOs in a human dermal fibroblast system. All studied oils showed strong and significant anti-proliferative effects against these cells. These oils demonstrated differential effects on protein molecules closely related to inflammation, immunomodulation, and tissue remodeling. Bergamot, cilantro, and spikenard EOs primarily exerted anti-inflammatory and tissue-remodeling effects, while helichrysum, ylang ylang, and Immortelle EOs primarily demonstrated tissue-remodeling effects. The data suggest that these EOs possess therapeutic potential; therefore, they might be possible effective adjunct therapies for a variety of conditions.

## Conflicts of interest

X.H., C.B., and N.S. are employees at dōTERRA, where the studied essential oils were manufactured.
