# Supplementary material for: Chemical composition analysis and in vitro biological activities of ten essential oils in human skin cells
Source: Biochim Open. 2017 Apr 26;5:1–7. doi: 10.1016/j.biopen.2017.04.001 (PMC5805555; doi:10.1016/j.biopen.2017.04.001)
Supplement: Supplementary file 1 [file mmc1.docx]

**Supplementary material**

Table S1. Glossary of biomarkers of the human dermal fibroblast system HDF3CGF used in the study

| **Readout** | **Description** |
| --- | --- |
| **CCL2/MCP-1** | MCP-1 system is a chemokine that mediates recruitment of monocytes and T cells into sites of inflammation. MCP-1 is categorized as an inflammation-related activity in the HDF3CGF system modeling Th1 inflammation involved in wound healing and matrix remodeling. |
| **CD106/VCAM-1** | VCAM-1 is a cell adhesion molecule that mediates adhesion of monocytes and T cells to endothelial cells. VCAM-1 is categorized as an inflammation-related activity. |
| **CD54/ICAM-1** | ICAM-1 is a cell adhesion molecule that mediates leukocyte-endothelial cell adhesion and leukocyte recruitment. ICAM-1 is categorized as an inflammation-related activity. |
| **Collagen I** | Collagen I is involved in tissue remodeling and fibrosis, and is the most common fibrillar collagen that is found in skin, bone, tendons and other connective tissues. Collagen I is categorized as a tissue remodeling-related activity. |
| **Collagen III** | Collagen III is an extracellular matrix protein and fibrillar collagen found in extensible connective tissues (skin, lung and vascular system) and is involved in cell adhesion, cell migration, tissue remodeling. Collagen III is categorized as a tissue remodeling-related activity. |
| **CXCL10/IP-10** | IP-10 is a chemokine that mediates T cell, monocyte and dendritic cell chemotaxis. IP-10 is categorized as an inflammation-related activity. |
| **CXCL11/I-TAC** | I-TAC is a chemokine that mediates T cell and monocyte chemotaxis. I-TAC is categorized as an inflammation-related activity. |
| **CXCL8/IL-8** | IL-8 is a chemokine that mediates neutrophil recruitment into acute inflammatory sites. IL-8 is categorized as an inflammation-related activity. |
| **CXCL9/MIG** | MIG is a chemokine that mediates T cell recruitment. MIG is categorized as an inflammation-related activity. |
| **EGFR** | EGFR is a cell surface receptor for epidermal growth factor involved in cell proliferation during development as well as tumor growth. EGFR is involved in Epithelial cell proliferation, epithelial cell differentiation keratinocyte proliferation, tissue remodeling. EGFR is categorized as a tissue remodeling-related activity. |
| **M-CSF** | M-CSF is a secreted and cell surface cytokine that mediates macrophage differentiation. M-CSF is categorized as an immune modulation-related activity. |
| **MMP-1** | MMP-1 is an interstitial collagenase that degrades collagens I, II and III and is involved in the process of tissue remodeling. MMP-1 is categorized as a tissue remodeling-related activity. |
| **PAI-I** | PAI-I is a serine proteinase inhibitor and inhibitor of tissue plasminogen activator (tPA) and urokinase (uPA) and is involved in tissue remodeling and fibrinolysis. PAI-I is categorized as a tissue remodeling-related activity. |
| **Proliferation_72hr** | Proliferation_72hr in the HDF3CGF system is a measure of dermal fibroblast proliferation which is important to the process of wound healing and fibrosis. |
| **SRB** | SRB is a measure of the total protein content of dermal fibroblasts. Cell viability of adherent cells is measured by Sulforhodamine B (SRB) staining, a method that determines cell density by measuring total protein content of test wells. |
| **TIMP-1** | TIMP-1 is a tissue inhibitor of matrix metalloprotease-7 (MMP-7) and other MMPs, and is involved in tissue remodeling, angiogenesis and fibrosis. TIMP-1 is categorized as a tissue remodeling-related activity. |
| **TIMP-2** | TIMP-2 is a tissue inhibitor of matrix metalloproteases and is involved in tissue remodeling, angiogenesis and fibrosis. TIMP-2 is categorized as a tissue remodeling-related activity. |
